# Usefulness of Airway Scope for intubation of infants with cleft lip and palate–comparison with Macintosh laryngoscope: a randomized controlled trial

**DOI:** 10.1186/s12871-018-0678-2

**Published:** 2019-01-12

**Authors:** Yoko Okumura, Masahiro Okuda, Aiji Sato Boku, Naoko Tachi, Mayumi Hashimoto, Tomio Yamada, Masahiro Yamada

**Affiliations:** Department of Anesthesiology, Aichi Gakuin University School of Dentistry, 2-11 Suemori-dori, Chikusaku, Nagoya 464-8651 Japan

**Keywords:** Airway scope, Macintosh laryngoscope, Infant, Intubation time

## Abstract

**Background:**

Airway Scope (AWS) with its plastic blade does not require a head-tilt or separate laryngoscopy to guide intubations. Therefore, we hypothesized that its use would reduce the intubation time (IT) and the frequency of airway complication events when compared with the use of Macintosh Laryngoscope (ML) for infants with cleft lip and palate (CLP).

**Methods:**

The parents of all patients provided written consents; we enrolled 40 infants with CLP (ASA-PS 1). After inducing general anesthesia using sevoflurane and rocuronium, we performed orotracheal intubations using either AWS (*n* = 20) or ML (n = 20), randomly. We define the duration between manual manipulation using cross finger for maximum mouth opening and the first raising motion of the chest following intubation by artificial ventilation as “IT;” further, the measured IT as primary outcomes. Airway complications were considered secondary outcomes. Moreover, we looked for associations between IT and the patient’s characteristics: extensive clefts, age, height, and weight. We used the Mann–Whitney test and Fisher’s exact probability test for statistical analysis; *p < 0.05* was considered as statistically significant.

**Results:**

The mean IT was 31.5 ± 8.3 s in AWS group and 26.4 ± 8.9 s in ML group. Statistical significant difference was not found in IT between the two groups. The IT of AWS group was statistically related to extensive clefts. Airway complications were detected in ML group.

**Conclusion:**

AWS could be useful for intubation of infants with CLP; it required IT similar to that required using ML, with a lower rate of airway complications.

**Trial registration:**

UMIN-CTR Registration number UMIN000024763.

Registered 8 November 2016.

## Background

It is recognized that intubation of infants is more difficult than that of adults [1] because infants have characteristics of macroglossia, i.e., both tongue and the epiglottis near the palate, a long and narrow epiglottis, and the large angle formed by trachea and vocal cords. Furthermore, intubation becomes difficult with craniofacial deformities or micrognathia [1]. Based on these factors, tracheal intubation is more difficult in the infants with cleft lip and palate (CLP) than in those without CLP [2, 3].

Ali et al [4] had compared intubation time for pediatric patients between pediatric Airtraq® and conventional Macintosh laryngoscope. They calculated the sample size on the bases of the mean outcome of intubation time required 30 s of the former and 40 s with a standard deviation of 8 s of the latter keeping α error 5% and power as 95% (1-β errors); therefore, the calculated sample size was 34 (17 in each group). Consequently, they concluded that Pediatric Airtaq® takes shorter time to intubate with less frequent complication than conventional laryngoscope in children.

In our hospital, Macintosh laryngoscope (ML) is the first alternative device for conventional intubation of infants with CLP. Alternatively, the blade of the Airway Scope (AWS) conforms to the upper airway and creates a groove for conducting a tracheal tube through the vocal cords, obviating the need for retroflexion of the head and spreading of the larynx. Therefore, AWS can potentially shorten intubation time (IT) compared to ML. Furthermore, AWS is unlikely to cause side effects as the blade is made of polycarbonate resin. However, it remains unclear as to which device is more useful for intubation of infants with CLP. We hypothesized that AWS would shorten IT and result in fewer side effects than ML.

## Methods

### Ethics approval and consent to participate

This study was approved by the Ethical Review Board of the Aichi Gakuin University School of Dentistry and was registered as a clinical trial with UMIN-CTR (No.000024763). The patients scheduled for cheiloplasty were hospitalized 2 days before the day of surgery based on specified rules of our hospital and received preanesthetic medical examination on that day. The patients were examined, and no systemic diseases or airway abnormalities were observed; therefore the anesthesiologist informed this research program, and written informed consent was obtained from the parents of the infant patients who agreed to participation in this study.

### Subjects

The inclusion criteria were patients scheduled for cheiloplasty between 9 November 2016 and 31 October 2017, aged 3–11 months, and American Society of Anesthesiologists physical status I. The exclusion criteria were patients whose parents declined to participate in this study, patients with medical history of airway abnormalities that needed tracheal intubation, or patients with cardiovascular complications.

**Fig. 1:**
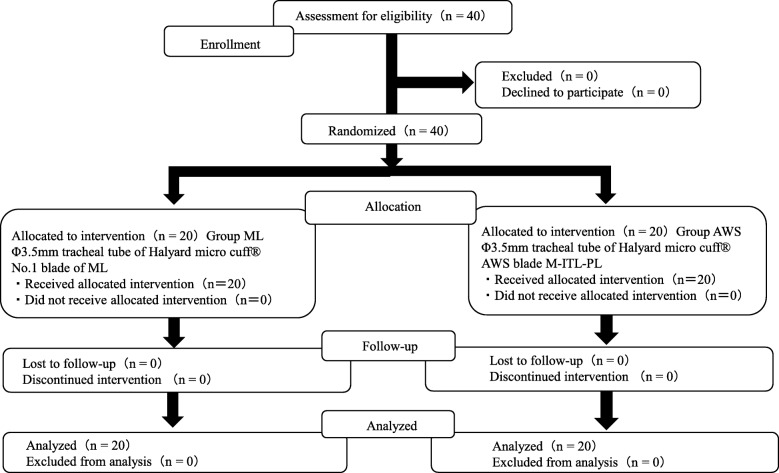
Consolidated Standards of Reporting Trials (CONSORT) recommended description of patient recruitment

We defined the duration between manual manipulation using cross finger for maximum mouth opening and the first raising motion of the chest following intubation by artificial ventilation as “IT;” further, we measured IT as the primary outcome. Based on the mean outcome of our pilot study including seven individuals for each group taken as 21 s in ML group and as 31 s in AWS group, we calculated effect size 1.00 using Cohen’s d first and subsequently the sample size keeping α error as 5%, and power of the study as 80% (1-β errors). the minimum sample size thus calculated was 34 (17 in each group) using G*Power 3.1.9.2. (http://www.gpower.hhu.de/). We allocated the patients to one of the two groups using the sealed envelope technique. The envelopes were opaque and stapled with the allocation card inside stored in one opaque storage box, which were prepared before the start of this study by our hospital clerk who was not related to this study. Finally, 40 cases of ASA-PS 1 CLP infants were finally included in this study because we prepared 40 envelopes to avoid numerical lack for this study. The investigating anesthesiologists included four expert anesthesiologists with experience of ML technique of more than 5 years and of using AWS few times in both adults and infants. They intubated the patients according to the allocation card which was handed by our hospital clerk on the morning of the operation. A Φ3.5 mm tracheal tube of Halyard micro cuff® was used in all patients as a first choice; the ML group patients were then intubated with No. 1 blade; the AWS group patients were intubated with AWS with NK PBLADE ITL-PL®. We recorded and analyzed the research data (Fig. 1).

### Evaluation parameters

We measured IT as primary outcomes. We also studied the visibility of the vocal cords, blade insertion, and tracheal tube insertion. Visibility of the vocal cords was evaluated by Cormack and Lehane grade and “quality of visual recognition of vocal cords,” which is a point system of evaluation of subjects on a scale of 0–100 according to the anesthesiologist. Difficulty in blade insertion was evaluated by the presence of backward or forward movement of the blade at the pharynx, number of blade insertions, and “blade insertion difficulty,” a point system of blade insertion operability evaluation on a scale of 0–100 according to the anesthesiologist. Difficulty of tracheal tube insertion was evaluated by the presence of changing head presentation, external compression of larynx, resizing of the tracheal tube if the Φ3.5 tube could not pass the glottis, and “difficulty of tracheal tube insertion,” which was a point system of evaluation of subjects from 0 to 100 according to the anesthesiologist. We also assessed the correlation between patient background and IT. Moreover, we recorded complications regarding intubation maneuver as secondary outcomes, which included desaturation (< 94%), bleeding from oral or pharyngeal tissue, esophageal intubation, and hoarseness after extubation.

### Statistical analysis

Characteristic of the patients, IT, numbers of attempts of intubation, visibility of vocal cords, and difficulty of blade insertion, and tracheal tube insertion were examined using the Student’s *t*-test or Mann–Whitney test. The items difficulty of tracheal tube insertion and frequency of occurrence of complications were examined using Fisher’s exact probability test. The correlation between patient background and IT was examined using Peason’s correlation coefficient test.

## Results

A total of 40 patients assessed for eligibility. All the patients were randomized for this study (Fig. 1). None of them excluded to follow up due to impossibility to intubate with the allocated method or postponement of the planned operation.

### Characteristic of the patients

There were no differences in the degree and region of the cleft, sex, age, height, or weight between the two treatment groups (Table 1).

**Table 1:** Preoperative patient characteristics and observation of difficulty in securing the respiratory tract

	ML				AWS				*p* value
Degree of cleft and Region of fissure (n)	CL		CLP		CL		CLP		0.53
	unilateral	bilateral	unilateral	bilateral	unilateral	bilateral	unilateral	bilateral	
	4	2	10	4	8	1	6	5	
Sex: Male or Female (n)	male		female		male		female		0.25
	12		8		15		5		
Age (month)	5.8 ± 1.7				5.1 ± 1.2				0.22
Height (cm)	65.1 ± 3.0				64.8 ± 2.8				0.71
Weight (kg)	7.2 ± 0.8				7.1 ± 0.6				0.73
Megaloglossia or hyperplasia of palatine tonsil (n)	0				0				1
Interincisor Distance (mm)	31.0 ± 3.9				29.2 ± 7.3				0.34
limitation of cervical spine mobility (< 90°) (n)	0				0				1
Difficulty of mask ventilation (n)	0				0				1

*CL* cleft lip, *CLP* cleft lip and palate

The suspicious observation of difficulty in securing the respiratory tract or spreading the larynx, defined as megaloglossia, hyperplasia of palatine tonsil, trismus, limitation of cervical spine mobility, or difficulty of mask ventilation were not detected preoperatively

### It

The average value of IT of the AWS group was 6 s greater than that of the ML group; however, significant difference was not detected in IT between the two groups (Table 2). Visibility of vocal cords was higher in the AWS group than in the ML group. The number of blade insertion attempts in the AWS group was greater than the ML group. There was no statistical difference in the number of cases with change in head presentation, need for external laryngeal pressure, or resizing the tracheal tube between both the groups (Table 2).

**Table 2:** IT and the factors affecting IT

		ML	AWS	*P* value
	Intubation time (sec)	26.4 ± 8.9	31.5 ± 8.3	0.07
Visibility of vocal cords	Cormack and Lehane grade median (IQR25%; IQR75%)	1(1; 1.5)	1(1; 1)	0.04
	Quality of visual recognition of vocal cords: good 0, bad 100 median (IQR25%; IQR75%)	0(0;10)	0(0; 20)	0.90
Difficulty of blade insertion	Number of cases with presence of backward or forward movement (n)	6	11	0.15
	Number of blade insertion: 1/2/3/4 (mean ± SD)	1	1.3 ± 0.5	0.04
	Difficulty of blade insertion: easy 0, dificult 100 median (IQR25%; IQR75%)	0(0; 10)	0(0; 20)	0.35
Difficulty of tracheal tube insertion	Number of cases with changing head presentation (n)	3	2	0.64
	Number of cases with external compression of larynx (n)	0	2	0.11
	Number of cases with resizing the tracheal tube (n)	0	1	0.32
	Difficulty of tracheal tube insertion: easy 0, dificult 100 median (IQR25%; IQR75%)	0(0; 22.5)	25(0; 40)	0.14

*:*p* < 0.05 (vs ML group)

### Correlation between patient characteristics and IT

There was a significant correlation between IT and degree of cleft in the AWS group; however, no correlation was found between IT and patient characteristics in the ML group (Table 3).

**Table 3:** Correlation between patient characteristics and IT in AWS group

	ML						AWS					
	correlation	*t* value	*p* value	t (0.975)	95% lower limit	95% upper limit	correlation	*t* value	*p* value	t (0.975)	95% lower limit	95% upper limit
Degree of cleft:	0.11	0.47	0.644	2.10	− 0.35	0.53	0.51	2.51	0.022	2.10	0.09	0.78
Region of fissure	−0.17	− 0.72	0.483	2.10	−0.57	0.30	0.40	1.87	0.077	2.10	−0.05	0.72
Age (month)	−0.17	−0.73	0.472	2.10	−0.57	0.29	−0.18	−0.77	0.452	2.10	−0.58	0.29
Height (cm)	0.04	0.18	0.862	2.10	−0.41	0.48	−0.04	− 0.18	0.858	2.10	−0.48	0.41
Weight(kg)	0.40	1.88	0.077	2.10	−0.047	0.72	−0.14	−0.59	0.563	2.10	−0.55	0.32

*:*p* < 0.05 (vs intubation time)

### Occurrence of complications

One case of bleeding from lip and three cases of bleeding from pharynx were observed in the ML group; however, none of these complications were detected in the AWS group. There was no correlation between occurrence of complications and IT in either group (Table 4).

**Table 4:** Occurrence of complications

	ML	AWS	*p* value
Desaturation (<94%) (n)	0	0	1
Bleeding from lips (n)	1	0	0.32
Bleeding from pharynx (n)	3	0	0.08
Bleeding from tongue (n)	0	0	1
Bleeding from palate (n)	0	0	1
Esophageal intubation (n)	0	0	1
Hoarseness after extubation (n)	1	1	1

## Discussion

Yu et al [5] conducted a meta-analysis of 14 clinical studies of infant intubations, and concluded that video laryngoscope improved visibility of vocal cords but increased IT and incidence of intubation failure compared with direct viewing laryngoscope using ML. In the current study, no significant difference was observed in IT between the AWS and ML groups. Therefore, these data indicate that AWS may be a viable substitution to ML for intubation of infants with CLP.

### Factors affecting IT

#### Visibility of vocal cords

Previous studies have attributed the difficulty of laryngoscopy in infants with CLP to young age [2], degree of cleft, and micrognathia [3]. In the current study, the Cormack and Lehane class of the ML group was statistically greater than that of the AWS group. However, quality of view was not statistically different between the groups. This result may be obtained when the Cormack and Lehane grade is less than III, which is an index of difficult intubation; this was not observed in any of the groups. Accordingly, the quality of view was appropriate for intubation in both AWS and ML; hence, the quality of view was not related to IT.

#### Difficulty of blade insertion

It has previously been reported that the tip of AWS blade may inadvertently access the esophagus rather than the trachea when inserted in the infant airway [6]. In the current study, the esophagus was seen first on screen following insertion into the pharynx in the AWS cases; therefore, the number of cases in which the blade was moved backward or forward was greater in the AWS group than in the ML group. Because of this, re-insertion was favorable to moving forward and backward to avoid injuring the pharynx in the AWS group; hence, the number of attempts of blade insertions was greater than that in the ML group. Moreover, the length of the AWS blade is 65 mm, which is longer than that of infant upper airway; therefore, it may have necessitated the increased instances of moving forward and backward in this group. Conversely, the attached documents for the Halyard micro cuff® recommends a Φ3.0 mm tracheal tube for infants aged < 8 months whose weight is > 3 kg, and thus the size of blade for neonates may be suitable for most of the patients in this study. In our hospital, a Φ3.5 mm tracheal tube of Halyard micro cuff® is generally the first choice for infants with CLP. Therefore, we chose the blade for pediatrics that was fit for the Φ3.5 mm tracheal tube. IT might be shortened using a Φ3.0 mm tracheal tube of Halyard micro cuff® and neonate blade of AWS (length is 12 mm shorter than that for pediatrics) because of reduction in the time required to determine the location of the esophagus and detect the vocal cord by moving backward.

#### Difficulty of tracheal tube insertion

**Fig. 2:**
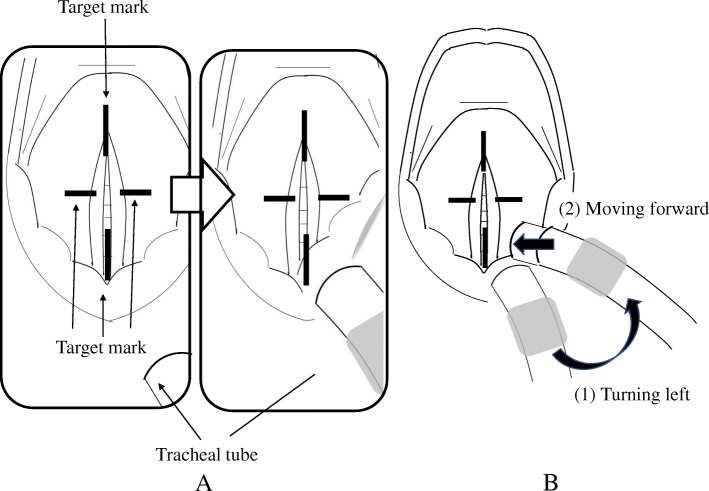
Schematic picture of tracheal intubation using AWS

Unless the AWS target mark coincides with the vocal cords at the monitor screen before progression of the tube, it is easy for the tip of the tube to inadvertently hit the right Rima glottides as it is progressed to the vocal cords after removing from the blade groove in infants [6] (Fig. 2a). Therefore, the tube needs to be turned left following removal of the tube from the blade groove prior to progressing to the vocal cords (Fig. 2b). “Difficulty of tracheal tube insertion” was not statistically different between the two groups in this study; however, this maneuver might be administered as needed with the progression of the tube; hence, average of IT of AWS may be longer than that of ML.

#### Correlation between patient characteristics and IT

In bilateral CLP, the premaxilla and cleft palate edge protrude to the oral cavity and narrow the space. This formation may limit controllability of the AWS blade, explaining the correlation of degree of cleft and IT in the AWS group.

### Complications

The AWS method has an increased risk of pharynx damage owing to the limited space between the blade and pharynx wall [7]. However, bleeding from upper airway was observed only in the ML group. The bleeding observed in the ML group was detected after fixing the tracheal tube with tapes on the lip. Nevertheless, anesthesiologists could watch the blade directly from the beginning of insertion to complete intubation when using ML. Therefore, the upper airway damage by ML may have been caused when the blade was removed from the pharynx or oral cavity. The number of cases with bleeding was not statistically different between the two groups; however, upper airway damage should be avoided, particularly in CLP surgery. More attention to upper airway mucosa is required not only at blade insertion but also at blade removal in ML even if an expert user of ML intubates, which might increase the IT time.

## Conclusion

AWS could be useful for the intubation of infants with CLP; it required IT similar to that required using ML with lower rate of airway complications.

## Abbreviations

AWS: Airway scope
CL: Cleft lip
CLP: Cleft lip and palate
IT: Intubation time
ML: Macintosh laryngoscope
